# Negative margin technique — a novel planning strategy to improve dose conformation in SBRT using dynamic conformal arc delivery

**DOI:** 10.1120/jacmp.v14i5.4283

**Published:** 2013-09-06

**Authors:** Siyong Kim, Taeho Kim, Stephen J. Ko, Christopher Serago, Ashley A. Smith, Laura A. Vallow, Jennifer L. Peterson, Rena Lee

**Affiliations:** ^1^ Department of Radiation Oncology Mayo Clinic Jacksonville FL USA; ^2^ Department of Radiation Oncology Ewha Womans University Seoul Korea

**Keywords:** field aperture margin, negative margin, dynamic conformal arc therapy, lung SBRT

## Abstract

The purpose of this study was to introduce a planning strategy for dynamic conformal arc therapy (DCAT), named negative margin technique (NMT), and evaluate its dosimetric gain in lung stereotactic body radiation therapy (SBRT). In DCAT, the field aperture is continuously conformed to the planning target volume (PTV) with an aperture margin (AM) to compensate for the penumbra effect with gantry rotation. It is a common belief the AM should be positive (or at least ‘zero'). However, the radial penumbra width becomes significantly wider because of continuously overlapped beams in arc delivery. Therefore, we hypothesize if the ‘negative margin’ is applied in the radial direction, it would improve the PTV dose conformation while reducing normal tissue dose. For verification, trial plans were made using the NMT and compared with ‘zero margin (ZM)’ plans for five lung SBRT cases representing different situations depending on the location of the PTV and organs at risk. All plans met 95% PTV coverage with the prescription dose and spared the spinal cord below the tolerance. Two conventional conformation indices (the ratio of prescription isodose volume to the PTV (CI100) and the ratio of 50% prescription isodose volume to the PTV (CI50)) and a modified conformation index were investigated. The maximum dose at 2 cm from the PTV $\left(D_{\text{max}‐2\text{cm}}\right)$ and the percent of lung volume receiving 20 Gy $\left(V_{20}\right)$ were also evaluated. Another planning simulation was performed with a total of ten randomly selected lung SBRT cases to mimic actual practice. In this simulation, optimization with ZM was first performed and further optimization using the NMT was processed for cases that could not meet a goal of ${\text{CI}}_{100}\;=\;1.2$ with the ZM optimization. In all cases, both the ${\text{CI}}_{100}$ and ${\text{CI}}_{50}$ values were significantly reduced (overall, 9.4% ± 4.1% and 5.9% ± 3.1% for ${\text{CI}}_{100}$ and ${\text{CI}}_{50}$, respectively). The modified conformation index values also showed similar improvement (overall, 10.1% ± 5.7% increase). Reduction of $D_{\text{max}‐2\text{cm}}$ was also observed in all cases (4.5% ± 2.2%). $V_{20}$ values decreased in all cases but one (5.7% ± 3.9%, excluding the increased case). In the random group simulation, it was possible to achieve the goal with just one NMT trial for five out of six cases that did not meet the goal in the ZM optimization. Interestingly, however, one case needed as many as six iterations to get the ${\text{CI}}_{100}\;=\;1.2$ goal. The NMT turned out to be an effective planning strategy that could bring significant improvement of dose conformation. The NMT can be easily implemented in most clinics with no prerequisite.

PACS number: 87.55.D‐

## I. INTRODUCTION

For the improvement of local control in small lesions in certain disease sites such as lung and liver, stereotactic body radiation therapy (SBRT) has been applied.^1^, ^2^, ^3^ Because of significantly larger fractional dose, SBRT requires high dose conformality, as well as precise beam delivery, and various beam delivery and planning techniques have been developed.^4^, ^5^, ^6^ Dynamic conformal arc therapy (DCAT), developed mainly for stereotactic radiosurgery (SRS), is capable of delivering conformal doses with efficiency for SBRT.^6^, ^7^, ^8^

Due to a significantly high ablative dose prescription compared to conventional therapy, dose conformation is extremely important in SBRT. For example, the Radiation Oncology Therapy Group (RTOG) 0915 protocol, a randomized phase II study comparing two SBRT fractionation schedules for medically inoperable patients with stage I peripheral non‐small cell lung cancer, requires a conformality index of smaller than 1.2 for the planning target volume (PTV).^(1)^

In this study, we introduce a planning strategy of DCAT for the improvement of dose conformation, and evaluate its dosimetric gain in lung SBRT.

## II. MATERIALS AND METHODS

### A. Theory/hypothesis

While the gantry rotates, in DCAT, the field aperture is continuously conformed to the planning target volume (PTV) in the beam's eye view (BEV) with an aperture margin (AM) to compensate for the penumbra effect. It is a common belief that the field aperture should be larger than or at least the same as the PTV to get adequate target coverage. However, it has been observed that the radial penumbra in the arc plane becomes significantly wider due to the continuous dose overlapping nature of the arc delivery. Thus, it may be hypothesized that if negative margin (NM) in the radial direction is allowed contrary to the common belief, it would improve the PTV dose conformation while reducing the surrounding normal tissue dose. This newly introduced method is named ‘negative margin technique (NMT)'.

### B. Study with a systematic group

#### B.1 Planning trials

**Figure 1:**
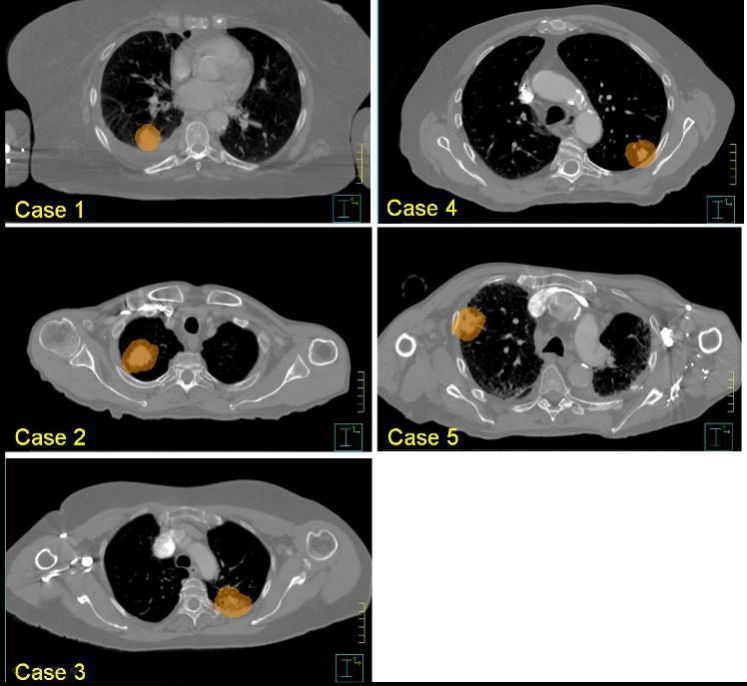
Axial plane at the isocenter level for each case in the systematic group: PTVs are in orange color wash (displayed in scales close each other for easy comparison).

For the verification of the hypothesis, planning simulation was performed with a total of five actual lung SBRT cases. Each of the five cases was systematically chosen to represent a distinguishable clinical situation, as shown in Fig. 1:

Case 1 — the PTV was relatively small and located with enough distance from each organ at risk (OAR); Case 2 — the PTV was relatively large and located in a moderate distance from the spinal cord; Case 3 — the PTV was located close to the spinal cord; Case 4 — the PTV was located at lateral‐posterior corner with relatively close distance to the skin; and Case 5 — the PTV was located too laterally, thus only partial arc was available due to collision problem.

For each case, two plans, one with ‘zero aperture margin’ (noted as ZM) and the other using negative aperture margin technique (noted as NMT), were obtained. Planning was carried out using a Philips Pinnacle (ver. 9) treatment planning system (TPS) (Philips Healthcare, Andover, MA) with a 6 MV photon beam of a Varian IX machine. Dose calculation was performed with heterogeneity correction using convolution‐superposition algorithm which showed minimal perturbation in small photon fields.^(9)^ All plans met two major planning objects — covering at least 95% of the PTV with the prescription dose (48 Gy in 4 fractions), and sparing the spinal cord below the tolerance (volumes receiving 20.8 and 13.6 Gy or more should be less than 0. 35 and 1.2 cc, respectively). Each PTV was obtained by adding 5 mm margin all around to its corresponding full‐breathing cycle‐based internal target volume (ITV). Information on internal breathing motion was obtained from a 10‐phase‐based 4D CT, while dose calculation was made on a free‐breathing CT dataset.

Depending on the situation, it is necessary to assign a nonuniform aperture margin around the PTV. However, the version of Pinnacle TPS used did not support assigning a nonuniform aperture margin. Thus, instead of entering aperture margin values, a pseudovolume (called pseudo‐PTV), initially generated by copying the PTV, was modified in six different directions (i.e., anterior, posterior, right, left, superior, and inferior direction) as needed, then the field aperture was conformed to the pseudo‐PTV.

As implied before, when DCAT is used within an axial coplane, dose penumbra in both the superior and inferior directions is steep and a positive aperture margin is necessary, as in the conventional approach. In this study, about 2 to 4 mm positive aperture margin was used in superior and inferior directions, while negative aperture margins were applied to other directions (i.e., anterior, posterior, right, and left direction). The amount of negative margin varied case to case. Clockwise rotation of gantry when seen from the table side was used for all arc beams. Gantry angle is 0° when it is heading toward the floor.

#### B.2 Evaluation

Two conventional conformation indices (CIs), the ratio of prescription isodose volume to the PTV $\left({\text{CI}}_{100}\right)$ and the ratio of 50% prescription isodose volume to the PTV $\left({\text{CI}}_{50}\right)$, were investigated. As described by RTOG,^(10)^ an ideal dose conformation makes a ${\text{CI}}_{100}$ equal to 1. When the irradiated volume is larger than the target volume, ${\text{CI}}_{100}$ is greater than 1, indicating inclusion of surrounding normal tissue within the volume having at least the prescription dose.

If the target volume is only partially irradiated to the necessary dose (i.e., prescription dose), it makes a ${\text{CI}}_{100}$ smaller than 1.

In addition to the conventional conformation indices, a modified conformation index (noted as ${M‐\text{CI}}_{100}$ in this study) suggested by Paddick^(11)^ was also investigated. ${M‐\text{CI}}_{100}$ is defined as:

(1) $$TV_{PIV^{2}}/\left(TV\times PIV\right)$$

where *TV* is target volume, *PIV* is prescription isodose volume, and ${\text{TV}}_{\text{PIV}}$ is the overlapped volume between *TV* and *PIV* (i.e., target volume having prescription dose). As can be noted in the equation, ${M‐\text{CI}}_{100}$ is either smaller or equal to 1. While ${\text{CI}}_{100}$ is subject to giving a false perfect score depending on the situation, ${M‐\text{CI}}_{100}$ is not.

Besides conformation indices, the maximum dose at 2 cm from the PTV as a percentage of the prescription dose $\left(D_{\text{max}‐2\text{cm}}\right)$ and the percent of lung volume receiving 20 Gy or more $\left(V_{20}\right)$ were evaluated.

### C. Study with a random group

To closely mimic actual practice, another planning simulation was performed with a total of ten randomly selected lung SBRT cases. In this simulation, optimization with zero aperture margin in radial direction was first performed and evaluated based on ${\text{CI}}_{100}$. A goal of ${\text{CI}}_{100}\;=\;1.2$ is routinely used in our clinic. Thus, if a case met this goal with zero aperture margin, no further optimization was processed. For other cases, however, optimization was continued until the goal was achieved and the number of iterations was recorded.

## III. RESULTS

### A. Systematic group study

Compared to the conventional zero margin plans, both ${\text{CI}}_{100}$ and ${\text{CI}}_{50}$ values decreased significantly in the NMT plans for all five cases (overall, 9.4% ± 4.1% reduction in ${\text{CI}}_{100}$ and 5.9% ± 3.1% reduction in ${\text{CI}}_{50}$). ${M‐\text{CI}}_{100}$ values also showed similar improvement (overall, 10.1% ± 5.7% increase). Note improvement means reduction in conventional CIs and increase in M‐CI. Table 1 summarizes detailed CI values for all cases. Other important parameters such as PTV volume, percent prescription isodose line chosen, beam arc ranges, and aperture margins used are also shown in the Table 1. In Case 1, for example, the PTV was $20.5\;{\text{cm}}^{3}$, the prescription isodose lines were 80% and 76% for the ZM and NMT, respectively; the aperture margins in the longitudinal direction were +3 mm for both the superior and inferior directions; the aperture margins in the radial direction were ‐1 mm for all directions (i.e., anterior, posterior, right, and left direction) for the full range of angles (i.e., from 181° to 180° clockwise); ${\text{CI}}_{100}$ values were 1.27 in the ZM plan and 1.16 in the NMT plan; the change of ${\text{CI}}_{100}$ from the ZM to the NMT was ‐8.7%; ${\text{CI}}_{50}$ values were 4.67 in the ZM plan and 4.21 in the NMt plan; the change of ${\text{CI}}_{50}$ from the ZM to the NMT was ‐9.9%; ${M‐\text{CI}}_{100}$ values were 0.72 in the ZM plan and 0.78 in the NMT plan; and the change of ${M‐\text{CI}}_{100}$ from the ZM to the NMT was 8.2%.

As shown in Table 1, for Cases 2 and 3 where the spinal cord was of concern, multiple partial arcs were used instead of one full arc. With multiple partial arcs it was possible to better optimize dose distributions by assigning different beam weights for different partial arcs (mainly to avoid irradiating the spinal cord over the tolerance). Having different beam weights for different partial arcs caused less symmetric dose distributions in the radial direction in the ZM plan. For this reason, negative aperture margins were applied for only two partial arcs (e.g., 261°‐330° arc and 81°‐150° arc in Case 2), and margins were nonuniform (e.g., ‐2 mm in anterior direction and ‐3 mm in posterior direction in Case 2) in the NMT plan.

**Table 1:** Comparison of conformation indices between conventional zero margin (ZM) plans and negative margin technique (NMT) plans for all tested cases in the systematic group

*Case*	$\text{PTV}\;({\text{cm}}^{3})$	*%Px Isodose (ZM/NMT)*	*AM SI (mm)*	*ARC Angle (°)*	*AM APRL (mm)*	${\text{CI}}_{100}\;(\text{ZM})$	${\text{CI}}_{100}\;(\text{NMT})$	$\Delta {\text{CI}}_{100}\;(\%)$	${\text{CI}}_{50}\;(\text{ZM})$	${\text{CI}}_{50}\;(\text{NMT})$	$\Delta {\text{CI}}_{50}\;(\%)$	${M‐\text{CI}}_{100}\;(\text{ZM})$	${M‐\text{CI}}_{100}\;(\text{NMT})$	$\Delta {I‐\text{CI}}_{100}\;(\%)$
1	20.5	80/76	S&I+3	181–180	‐1	1.27	1.16	‐8.7	4.67	4.21	‐9.9	0.72	0.78	8.2
2	52.3	76/75	S&I+2	181–260	NC	1.18	1.13	‐4.2	4.54	4.40	‐3.1	0.78	0.80	3.3
				261–330	A‐2, P‐3									
				331–80	NC									
				81–150	A‐2, P‐3									
				151–180	NC									
3	45.4	80/77	S&I+2	181–220	NC	1.34	1.19	‐11.2	5.11	4.73	‐7.4	0.68	0.77	13.5
				221–316	A‐2, P‐5									
				317–46	NC									
				47–136	A‐2, P‐5									
				137–180	NC									
4	30.8	72.5/77	S+3, I+4	181–180	A&R+3, P&L‐3	1.38	1.17	‐15.2	5.35	5.21	‐2.6	0.66	0.77	17.8
5	32.3	80/80	S&I+3	181–90	A‐l, R‐l	1.30	1.20	‐7.7	4.81	4.49	‐6.7	0.71	0.76	7.5
							Mean	‐9.4		Mean	‐5.9		Mean	10.1
							SD	4.1		SD	3.1		SD	5.7

%Px: = percent prescription; AM = aperture margin; S, I, A, P, R, and L = superior, inferior, anterior, posterior, right, and left directions (e.g., A‐2 indicates ‐2 mm margin in anterior direction); NC = no margin change; ACI = change of Cl from ZM to NMT in % = [CI(NMT) / CI(ZM) ‐ 1] × 100.

Because the PTV was located close to the skin in Case 4, a noticeably asymmetrical dose distribution with hotter dose skewed toward the skin was obtained in the ZM plan. To compensate for such severe dose asymmetry, positive aperture margins (+3 mm) were used for both the anterior and right directions, while negative aperture margins (‐3 mm) were chosen for both the posterior and left directions, which brought significant gain in ${\text{CI}}_{100}$ (i.e., 15.2% reduction from the ZM to NMT plan).

In Case 5, the PTV was placed too laterally to have a full arc without collision. Thus, a partial arc beam from 181° to 90° clockwise was used and it caused more generous dose distributions in the anterior and right directions, which then resulted in necessitating negative margins in both the anterior and right directions.

Comparisons of $D_{\text{max}-2\text{cm}}$ (the maximum dose at 2 cm from the PTV as a percentage of the prescription dose) and $V_{20}$ (the percent of lung volume receiving 20 Gy) between the ZM and NMT plan were summarized in Table 2. As shown, reduction in the $D_{\text{max}-2\text{cm}}$ was observed in all cases (average −4.5% ± 2.2% change). Except in Case 4, the values of $V_{20}$ also decreased (average −5.7% ± 3.9% change, excluding Case 4). In Case 4, the PTV was located close to the posterior side of the lung and the dose distribution was tilted towards the back in the ZM plan. With the margins applied in the NMT plan, the dose distribution was moved towards the anterior side to cover the PTV better, resulting in an increase of $V_{20}$ (11.7% change). However, even the increased $V_{20}$ (i.e., 2.2%) was clinically insignificant.

**Table 2:** Comparison of the maximum dose at 2 cm from the PTV as a percentage of the prescription dose $\left(D_{\text{max}‐2\text{cm}}\right)$ and the percent of lung volume receiving 20 Gy $\left(V_{20}\right)$ between conventional zero margin (ZM) plans and negative margin technique (NMT) plans for all tested cases in the systematic group

*Case*	$D_{\text{max}‐2\text{cm}}\;(\text{ZM})$	$D_{\text{max}‐2\text{cm}}\;(\text{NMT})$	$\Delta D_{\text{max}‐2\text{cm}}\;(\%)$	$V_{20}\;(\text{ZM})$	$V_{20}\;(\text{NMT})$	$\Delta V_{20}\;(\%)$
1	49.2	47.3	‐3.9	2.88	2.64	‐8.3
2	64.4	63.0	‐2.2	3.80	3.74	‐1.6
3	76.8	70.7	‐7.9	4.72	4.27	‐9.5
4	57.9	54.8	‐5.4	1.97	2.20	11.7
5	59.3	57.4	‐3.2	3.39	3.28	‐3.2
			Mean ‐4.5		Mean	‐2.2
			SD 2.2		SD	8.4
					Mean w/o Case 4	‐5.7
					SD w/o Case 4	3.9

$\Delta_{\text{max}‐2\text{cm}}\;=\;$ change of $\Delta D_{\text{max}‐2\text{cm}}$ form ZM to $\text{NMT}\;=\;[D_{\text{max}‐2\text{cm}}\;(\text{NMT})/D_{\text{max}‐2\text{cm}}(\text{Zm})\;‐\;1]\;\times \;100$; $\Delta V_{20}\;=\;\text{change of}\;\Delta V_{20}$ from ZM to $\text{NMT}\;=\;[V_{20}\;(\text{NMT})\;/V_{20}\;(\text{ZM})\;‐\;1]\;\times \;100$.

**Figure 2:**
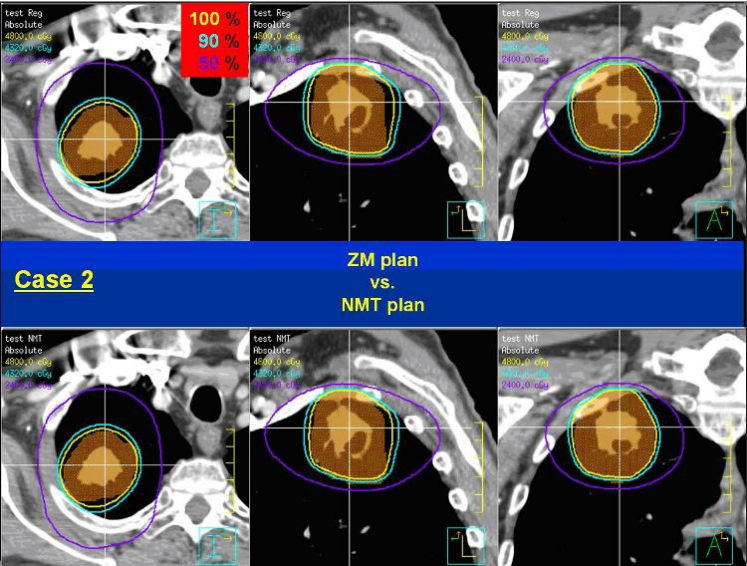
Dose distribution comparison between the ZM and NMT plan for Case 2: ZM plan (top), NMT plan (bottom), axial plane (left), sagittal plane (middle), and coronal plane (right).

**Figure 3:**
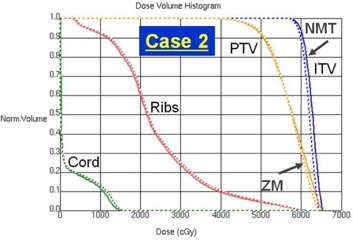
Dose‐volume histogram (DVH) comparison between the ZM and NMT plan for Case 2: ZM plan (dashed line), NMT plan (solid line), ITV (blue), PTV (orange), ribs (red), and spinal cord (green).

Figure 2 shows a comparison of the dose distributions for the ZM and NMT plans in Case 2 where the improvement of dose conformality is the least $\left(\Delta {\text{CI}}_{100}\;=\;‐4.2\%\right)$ among all tested cases. The top row is for the ZM plan and the bottom for the NMT plan. From the left, the axial, sagittal, and coronal planes are displayed. It's not huge, but a clear improvement in dose conformality to the target can be observed in the NMT plan, especially in both the axial and sagittal planes. The dose‐volume histograms (DVHs) of the same case are shown in Fig. 3, where dashed lines are for the ZM plan and solid lines for the NMT plan. Blue, orange, red, and green color indicates the ITV, PTV, ribs, and spinal cord, respectively. Overall, as can be seen, dose to the target (e.g., the ITV and PTV) is higher in the NMT plan, while dose to the OARs (i.e., the ribs and spinal cord) is lower, implying superior dosimetric quality of the NMT plan over the ZM plan in terms of dose conformation. The similar trend of improvement (in larger amount) was observed in other cases, except in Case 4.

**Figure 4:**
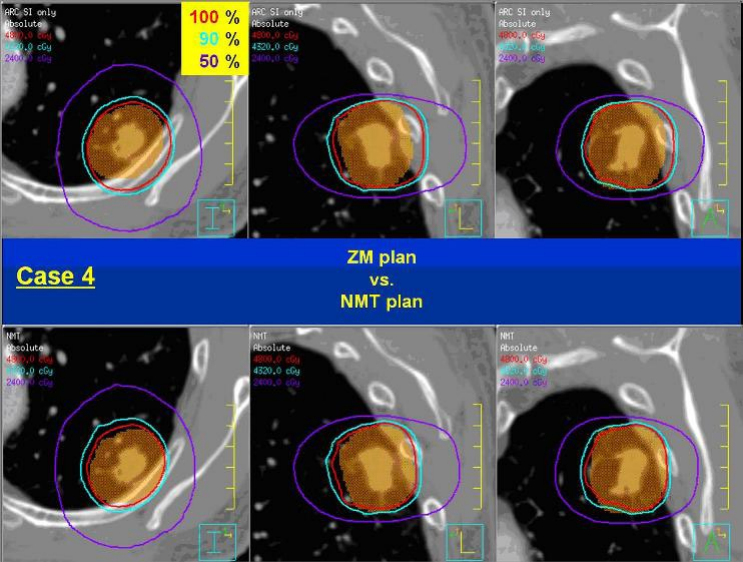
Dose distribution comparison between the ZM and NMT plan for Case 4: ZM plan (top), NMT plan (bottom), axial plane (left), sagittal plane (middle), and coronal plane (right).

**Figure 5:**
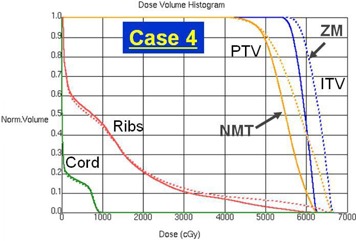
Dose‐volume histogram (DVH) comparison between the ZM and NMT plan for Case 4: ZM plan (dashed line), NMT plan (solid line), ITV (blue), PTV (orange), ribs (red), and spinal cord (green).

As noted before, the PTV was located close to the posterior side of the lung in Case 4, which caused severe dose asymmetry in the ZM plan. In the NMT plan, the whole dose distribution was moved anteriorly to cover the PTV better and such significant change can be observed in Fig. 4, which shows a comparison of the dose distributions between the ZM and NMT plan (plotted with the same format as Fig. 2). As can be seen, excessive target coverage extruding even to the surrounding normal tissue in the posterior direction is observed in the ZM plan, while slightly tight coverage is made in the anterior side. However, a more conformal dose distribution similar to the other cases is observed in the NMT plan. Figure 5 shows the DVHs in the same format as Fig. 3 for Case 4. Contrary to other cases, dose to the target is higher in the ZM plan. This was due to the fact that the planner had to choose a very low prescription isodose line (i.e., 72.5%) to meet the plan objective under the significantly asymmetric dose distribution. Such issue was resolved and a higher level of isodose line (i.e., 77%) was chosen in the NMT plan. In addition, dose to the ribs is also significantly lower in the NMT plan, clearly manifesting the benefit of negative margin technique.

### B. Random group study

Six out of ten cases showed ${\text{CI}}_{100}$ of higher than 1.2 in the ZM optimization. Further optimization using the NMT for these cases brought significant improvement (overall, 8.9% ± 2.3% reduction in ${\text{CI}}_{100},8.7\%\;\pm \;2.4\%$ reduction in ${\text{CI}}_{50}$, and 9.8% ± 2.1% increase in ${M‐\text{CI}}_{100}$), resulting in the achievement of planning goal of ${\text{CI}}_{100}\;=\;1.2$ or less for every case. Detailed values are summarized in Table 3. To be distinguished from the systematic group, cases in the random group are noted with letter ‘R’ in the front of each case number (e.g., Case R1, Case R2, and so on). Regarding the number of iterations, it was possible to achieve the goal with just one NMT trial for all cases, except Case R8. However, Case R8 needed as many as six iterations to get the ${\text{CI}}_{100}$ of 1.2.

**Table 3:** Conformation index improvement using NMT and the number of iterations needed to achieve ${\text{CI}}_{100}=\;1.2$ or less in the random group (each case is noted with both a letter ‘R’ and number to be discernible from the systematic group)

*Case*	*PTV*(cm3)	*%Px Isodose (ZM/NMT)*	${\text{CI}}_{100}$ *(ZM)*	${\text{CI}}_{100}$ *(NMT)*	$\Delta {\text{CI}}_{100}(\%)$	${\text{CI}}_{50}$ *(ZM)*	${\text{CI}}_{50}$ *(NMT)*	$\Delta {\text{CI}}_{50}\;(\%)$	${M‐\text{CI}}_{100\;(\text{ZM})}$	${M‐\text{CI}}_{100}\;(\text{NMT})$	$\Delta {M‐\text{CI}}_{100}(\%)$	*Iteration (#)*
**R1**	**16.1**	**80/77**	**1.30**	**1.14**	**‐11.9**	**6.07**	**5.38**	**‐11.4**	**0.70**	**0.79**	**12.8**	**1**
R2	20.2	81/NA	1.19	NA	NA	5.30	NA	NA	0.76	NA	NA	NA
R3	81.0	85.5/NA	1.17	NA	NA	4.74	NA	NA	0.78	NA	NA	NA
R4	25.3	83/NA	1.15	NA	NA	4.61	NA	NA	0.79	NA	NA	NA
**R5**	**40.8**	**78/77**	**1.32**	**1.19**	**‐10.2**	**5.21**	**4.75**	**‐8.9**	**0.69**	**0.76**	**10.6**	**1**
**R6**	**121.5**	**80/77.5**	**1.23**	**1.15**	**‐6.8**	**4.87**	**4.56**	**‐6.3**	**0.73**	**0.79**	**7.8**	**1**
**R7**	**15.3**	**82/81**	**1.22**	**1.12**	**‐8.2**	**4.59**	**4.19**	**‐8.8**	**0.74**	**0.82**	**9.7**	**1**
**R8**	**24.4**	**75.5/71.5**	**1.33**	**1.20**	**‐10.2**	**6.52**	**5.78**	**‐11.3**	**0.74**	**0.82**	**11.0**	**6**
	R9	87.4	82/NA	1.17	NA	NA	4.15	NA	NA	0.78	NA	NA
**R10**	**30.8**	**76.5/74**	**1.27**	**1.19**	**‐6.1**	**4.95**	**4.68**	**‐5.6**	**0.71**	**0.76**	**7.1**	**1**
				Mean	‐8.9		Mean	‐8.7		Mean	9.8	1.8
				SD	2.3		SD	2.4		SD	2.1	2.0

Note: Cases having higher than C1100 = 1.2 in ZM optimization are in bold.

%Px = percent prescription; ÄCI = change of CI from ZM to NMT in % = [CI(NMT) / CI(ZM)‐ 1] × 100; NA = not applicable.

## IV. DISCUSSION

One of the advantages of the DCAT in coplanar geometry is a reduced treatment time compared to static 3D conformal therapy consisting of multiple noncoplanar beams. With reduced treatment time, patients may move less during treatment^(12)^ and feel more comfortable. In our clinic, most patients are elderly and/or have a poor performance status; thus, they are expected to benefit from reducing treatment time. Reduction of treatment time can also improve machine utility and health‐care economics in principle. Coplanar 3D conformal therapy with multiple gantry angles (mostly more than seven) may provide a similar dose distribution as DCAT surrounding the target with just slightly longer treatment time. However, static beam technique is more susceptible to increased skin dose in principle. Another advantage of the coplanar DCAT is that it is not subject to potential increase of mechanical uncertainty related to couch rotation for noncoplanar beam arrangement. However, there are disadvantages in the coplanar DCAT and the most important one is that the range of beam angle selection is limited to a single plane, which may hinder getting desired dose conformality, depending on the situation. The negative margin technique introduced in this study was able to compensate for such limitation by significantly improving dose conformality.

In principle, similar gains as the NMT brings to DCAT plans can be achieved by volumetric‐modulation arc therapy (VMAT). However, compared to DCAT there exists more uncertainty in an intensity modulation technique due to inevitable interplay effect between target motion and beam aperture motion, as demonstrated by Berbeco et al.,^(13)^ especially for hypofractionation treatment which is usual in SBRT. In addition, VMAT costs more than DCAT in terms of both billing and man‐power utility in the current health‐care system. Therefore, based on those facts (DCAT is more robust and economical), DCAT is the first choice in our clinic, and VMAT is used only when it is difficult to obtain an acceptable dose distribution with DCAT, as in the case when either multiple targets exist closely or critical organs are located too close to the target.

When a target is located very close to skin, there is a high probability that dose to the skin exceeds the accepted tolerance, increasing the risk of skin complications. As illustrated in Case 4, the NMT enables manipulating the dose distribution in a certain degree to reduce skin dose in such cases.

The PTV, in Case 5, was placed laterally and it was not possible to have a full arc due to the issue of gantry collision with either the patient or table when the isocenter was at the center of the PTV. In such a situation, an alternative solution is to set up the isocenter centrally outside the PTV and use off‐axis fields to get full arc geometry without collision problems. However, in principle, this approach is more susceptible to mechanical uncertainties, such as collimator angle and gantry angle error. Thus, treating with partial arc geometry with the isocenter at the PTV center is preferred in our clinic. It is inevitable to have more asymmetric dose distribution in that case than usual, and the NMT can mitigate such an issue, as demonstrated in this study.

It is generally true that beam margin is correlated with dose heterogeneity. More specifically, lower prescription isodose line (PIL) is expected with tighter margin. Except Case 4, which represents a totally different situation from others as explained before, selected PILs in the NMT plans are lower than those in the ZM plans for all cases. However, as can be seen in 1, 3, the amount of PIL change does not seem significant (i.e., less than 5%) compared to that of conformation index change (i.e., reaching up to over 12%). We believe this is related to the shape of dose distribution (dose gradient) near the point where the PIL is selected. The PILs in this study are at about 80% for the ZM plans where dose gradient is not that steep. Thus, margin change in few mm would not cause significant PIL change which is directly related to dose heterogeneity.

The negative margin needed in Case 3 reached up to 5 mm in posterior direction for two partial arcs. Although it seems quite large, the target can still get necessary dose because the rest of the arcs bring enough dose in posterior direction.

During the plan optimization process in this study, the negative margins were determined by trial and error. Thus, the margin values might not be fully optimized. It is considered that more optimal values could be obtained if more time and endeavor were invested. Based on the random group study, it seems possible to get satisfactory optimization with reasonable effort for most cases. Compared to other cases in the random group study, however, Case R8 required significantly larger number of iterations (six vs. one) to achieve the ${\text{CI}}_{100}\;=\;1.2$ goal. It is interesting to note that for Case R8, the ${\text{CI}}_{100}$ values were 1.33, 1.25, 1.23, 1.21, 1.21, 1.21, and 1.2 in the order of iteration from zero to six. This implies there are chances, although not frequent, that a huge effort is necessary to get fine conformation improvement. We believe if the NMT is incorporated into a treatment planning system as a plan optimization algorithm, it would provide more consistent and optimal margins, as well as make the process simpler. Dose conformation with DCAT is heavily related to the dose gradient in the penumbra region, especially at the isodose point chosen for dose prescription. Dose gradient is a function of many parameters such as beam quality, field size, and irradiation material. Thus, it would be useful to evaluate dose distribution characteristics according to such parameters to properly develop the NMT optimization algorithm for a TPS.

In this study, we mainly focused on lung SBRT but, in principle, the NMT can be applied to some other sites such as liver. However, both the amount of negative margin needed and the gain realized may be different, and independent analyses would be needed for the expansion of NMT to other disease sites.

## V. CONCLUSIONS

Compared to the conventional zero margin plans, the level of dose conformality was significantly better in the negative margin technique plans. Based on the results of plan simulations, NMT is an effective planning strategy that could bring significant improvement of dose conformation. When the NMT is applied, planners are expected to achieve plan objectives with coplanar field(s) only, which then would result in efficient beam delivery with less mechanical uncertainty. The NMT can be easily implemented in most clinics with no prerequisite.

## ACKNOWLEDGMENTS

This study was in part supported by the Industrial Strategic Technology Development Program (10035527) funded by the Ministry of Knowledge Economy (MKE, Korea).
